# Driving behavior and safety analysis at OSMS section for merged, one-way freeway based on simulated driving safety analysis of driving behaviour

**DOI:** 10.1371/journal.pone.0228238

**Published:** 2020-02-13

**Authors:** Nengchao Lyu, Yue Cao, Chaozhong Wu, Alieu Freddie Thomas, Xu Wang

**Affiliations:** 1 Intelligent Transportation Systems Research Center, Wuhan University of Technology, Wuhan, China; 2 School of Transportation, Shandong University, Jinan, China; Tongii University, CHINA

## Abstract

In order to study driving performance at the opening section of median strip (hereafter OSMS) on the freeway capacity expansion project, this study separately controlled 9 different simulated experimental scenarios of OSMS length and freeway traffic flow. 25 participants were recruited to perform 225 simulated driving tests using the driving simulator, and the analysis of variance (ANOVA) was used to analyze the driving characteristics which can represent the safety context. The results show that the safety parameters of driving are different when the length of OSMS and the traffic flow are different. When the traffic flow is low or moderate, the OSMS length can significantly affect the speed of the vehicle and the maximum values of time to collision. The higher the traffic flow, the smaller the minimum values of time headway. As the length of the OSMS decreases, the vehicles are more generally concentrated at the end of the opening area with the minimum values of time headway. The study also found that when the traffic volume is high, the impact of the OSMS length on driving performance will be weakened. In addition, the OSMS length and the traffic flow have little impact on driving comfort. Additionally, when the traffic flow is low or moderate, the opening length can significantly affect the driving behavior and safety of the vehicle. However, when the traffic volume is high, the impact of the opening length on them will be relatively weakened to some extent. Therefore, it is advised that in the case of freeways with large traffic volume, merely extending the length of the opening section does not necessarily optimize safety. Rather, the actual traffic density of the road should be carefully considered before a design length is adopted.

## 1. Introduction

In a bid to keep people and goods moving, traffic engineers are continuously challenged with coming up with innovative strategies to respond to increasing capacity demand on the transport infrastructure. A special case of such strategies has to do with an attempt to increase the capacity of a two-way flow freeway (with each direction of flow separated by a median strip) into a one-way flow freeway. In implementing this strategy, since a complete removal of the median strip is technically and economically imprudent, a key concern, has to do with the proper design of the optimal length of the opening section of the median strip that facilitates merging and diverging between the two sections of the freeway. This alteration turns the original ordinary two-way road into a one-way road, but with the median strip still in place-thereby creating an unfamiliar driving environment around the OSMS. The geometric difference between normal merging and diversion areas as usually seen around ramps and the merging and diversion area on OSMS in this context is that the end of the acceleration lane of the normal merging area is merged with the main lane, while the acceleration lane of the merging area of the OSMS zone is independent. The same is true for the diversion area. The opening length of the median strip is an important factor for ensuring safe driving around the OSMS zones. The conversion of two-way flow freeways into One-way roads with median strip separation belt, is a new and unique form of engineering challenge in Chinese freeway, and few studies have been conducted on the design of the opening length with regards to safety implications. In terms of theoretical research, earlier studies have explored the safety of such separation belts from the perspectives of interlacing theory, acceptable gap theory and queuing theory [1]. Further research has used the same method to further study the length and position of the OSMS [2] [3]. The above study theoretically analyzed the safety of different lengths of the OSMS. Subsequent studies also studied the recommended values for the OSMS length for different design speeds, median strip widths and cross slopes by driving characteristics of the vehicle at the center of the separation zone [4].

The length of the OSMS of the one-way road involves economic and safety issues. Increasing the opening length incurs more financial costs. Drivers often exhibit different driving behaviors under different rates of traffic flows and opening lengths. A clear understanding of how to design the length of the opening for different traffic volumes is yet to be established. However, there are few studies on the driving behavior at the OSMS on road capacity expansion project with one-directional flow freeway; and especially so, the safety quantitative analysis by safety surrogate indicator is currently rare. Proven simulated driving technology offers the potential for quantitative safety assessment of this new type of traffic scenario.

In order to identify the traffic accident risk factors at the OSMS at the one-way expressway, and to provide a basis for the design of the length of the opening section, it is necessary to study the driving behavior of the driver at the merging area of the OSMS. The main purpose of this work is to study the traffic safety characteristics of the interaction between the OSMS zone and the traffic flow. This study used 225 valid simulated driving test data to analyze the driving safety at the OSMS zone of the interchange entrance and exit area through safety surrogate indicators. According to different opening lengths and traffic volumes, nine simulated road scenes were designed, and the driving behavior data of driver groups in different types of scenes were statistically analyzed, including lane change point, vehicle speed, maximum deceleration, the minimum value of time headway, and the maximum value of reciprocal time to collision (TTC_i_). The main results are as follows.

(1) It is necessary to design different lengths of median strip openings depending on the traffic flow on the freeway.

(2) With regards to traffic volumes, the results show that when the traffic volume is at low or moderate levels, the opening length significantly affects driving behavior resulting in increased risks.

(3) At high traffic volumes, the impact of the opening length on driving behavior weakens to some extent.

(4) It is recommended that in the case of large traffic volume on the freeway, merely extending the length of the opening section does not necessarily increase the safety of the driving environment. Rather, the average operating traffic volume of the road should be carefully considered before a design length is adopted.

The study provides the basis for the design of the opening length of the OSMS in the freeway merging and diverging area and can serve as a good reference.

## 2. Literature review

In essence, this study, investigates a unique safety challenge that exists in the merging and diversion area on one-way freeway converted from an erstwhile two-way freeway. Although there is no specific research reference for this particular situation, there have been some related studies on driving behavior and traffic safety in the merging and diversion area of the expressway. Studies have shown that the accident rate in the merging and diversion zone on the expressway is the highest [5] [6]. The research on the driving behavior characteristics of the merging and the diverging area started earlier. The main research results include the speed of the vehicle, the lane change behavior and the distance headway. Golob conducted a survey on the highway entrance and exit ramps and their affected areas, studied the time characteristics of traffic conflicts in expressway interchanges, and analyzed the traffic flow interlacing and safety characteristics of the ramps and their connected parts [7]. Lyu [8] used field operational test data from 89 driving instances to analyze the driving behavior of drivers with different gender and experience in the deceleration area of freeway, which proves that different drivers have different driving characteristics. Gong [9] studied the distribution of vehicles in each lane with different lane restrictions, and fitted the statistical distribution of headway of the vehicle on the main lane and deceleration lane. In order to study the driving behavior characteristics of the merging and the diverging area, based on the research of *Highway Capacity Manual* on the capacity interchanges on freeway [10], Liu [11] proposed a continuous traffic flow model for the merging and diverging area. Jetto [12] studied the time required for the vehicles in the merging and diverging area to change lanes to the target lanes under different conditions, and proposed that the vehicle has two states in the process of diversion and confluence, namely the limit state and the safety state. In order to keep the vehicle in a safe state during the process, the critical length required for the vehicle is calculated according to the traffic flow density. This research shows that in the merging and diverging area, the safe state required for the vehicle to change lanes to the target lane in the process of diversion and confluence is closely related to traffic flow. Therefore, traffic flow is an important factor when studying the opening length required for safe lane change. Westphal [13] carried out a measured analysis of the traffic volume in the merging area of the freeway, established a traffic capacity model of the entrance ramp of merging area, and concluded that the vehicles of the ramp had influence on the vehicles of the main line. By studying the acceleration lane length and driving behavior. Wattleworth [14] found that the shorter the distance between the vehicle and the end of the acceleration lane, the stronger the lane change intention of the driver and the more likely to change lanes on shorter gaps. Michaels [15] established a model of convergence point location by analyzing the angle variation between the merged vehicle of the ramp and the rear vehicle from the main line. Feng [16] and Zhu [17] studied the impacts of vehicles’ lateral control modes on asphalt pavement performance, and a framework was proposed for different conditions.

Regarding the research methods of traffic safety in the merging area, most of the research mainly used the theoretical model to analyze traffic safety, and most focused on the relationship between traffic accidents and traffic flow. At present, the typical methods include accident evaluation method, fuzzy comprehensive evaluation method, and evaluation of driver's physiological and psychological indicators. Zeng [18] used the accident assessment method to analyze the collision rate on the interchange through accident data from a freeway in Florida for three years. Sarhan [19] used regression analysis to study the relationship between geometric parameters and traffic flow characteristics of 26 interchanges, and used the accident data to establish a negative binomial distribution model to analyze the relationship between geometric parameters of road and traffic flow and traffic accidents. Kita [20] analyzed the critical clearance selection of vehicles in the merging area of interchange, and the results show that the length of acceleration lane has a certain influence on the driver's choice of confluence gap. Hidas [21] proposed a microscopic model based on traffic congestion and traffic accidents, and analyzed the lane changing behavior of vehicles in the merging area in terms of vehicle speed, acceptable clearance, traffic conflict; including free lane change and forced lane change. It is common to study the traffic operation in the merging and diverging area by using simulation and measured data. Zeng [22] analyzed freeway crash severity using a Bayesian spatial generalized ordered logit model with conditional autoregressive priors. Barnes [23] predicted the traffic flow in the merging and diverging area by using the micro simulation model, FRESIM. Jolovic [24] used the macro-calculation software FREEVAL and the micro-simulation software VISSIM to study the traffic flow state in the merging area respectively, and analyzed the results. Calvi [25] used a driving simulator to study the driving behavior characteristics in the deceleration lane of the diverging area. The results show that the actual driving behavior characteristics are significantly different from the model parameters commonly used in the design. Lu [26] used a micro-simulation model to simulate the traffic operation of entrances and exits on freeway, and used actual data for verification and received good research results. Michalopoulos [27] developed a freeway simulation program and a new module to study the merging and diverging area of freeway based on observation data. Zeng [28] [29] used a multivariate random-parameters Tobit model for analyzing highway crash rates by injury severity for traffic safety analysis. Feng [30] used unbalanced panel data mixed logit model and real-time big data to analyze hourly crash likelihood.

Through the above analysis, it is found that the existing models and assumptions have the following problems. The existing models of merging area on freeway mainly study geometry parameters of road. Moreover, these models are mainly based on theoretical analysis and observation data, and it is impossible to further explore the microscopic driving characteristics of the merging area, such as the time headway and time to collision of the confluent vehicles, which are highly related to traffic safety [31] [32].

With the development of driving simulation technology and the innovation of data analysis technology, it is possible to study the vehicle driving characteristics under the new situation of the merging area. Unlike the common merging area, the new merging area proposed in this study is the confluence area between two main roads separated by a median strip but now having the same direction of vehicular flow which was hitherto opposite. However, the essence of convergence is still the same. Vehicles not only have to face the task of changing lanes to the target road in time, but also need to execute safe acceleration in the process of convergence so as to keep the speed of vehicles on the target road in line with that of vehicles on the target road. Based on the above considerations, this study analyzes the influence of the opening length of the median strip and the traffic flow on the driving characteristics based on the results of the simulated driving test, studies the corresponding safety substitution parameters, and evaluates the driving safety and comfort of different opening length and traffic volumes. The analysis of the traffic safety characteristics of the interaction between the OSMS zone and the traffic flow can be realized by studying the relationship between the opening length of the median strip and the traffic flow during traffic operations. Through the above research, the existing driving model of merging area can be improved, and the design and traffic management can also be supported by the corresponding theory.

## 3. Methodology

### 3.1 Research scenario and road model

**Fig 1:**
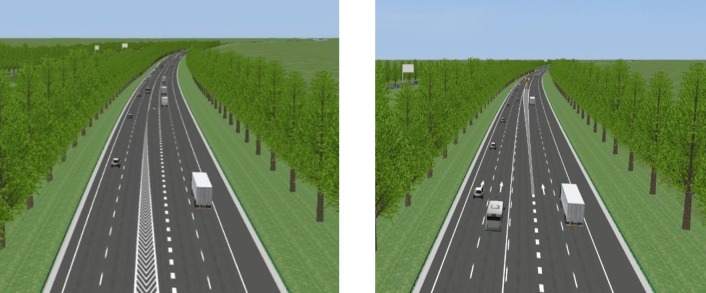
The road model at the beginning and end of the opening.

In China, most of the previous freeways were initially four lanes, which were later widened to eight lanes. Therefore, the modified one-way freeway is a four-lane road (half of the eight lanes).On removal of the median strip, an additional lane is created, which is the third lane (the center of the road) in Fig 1. Therefore, the expansion of four lanes to five lanes has become typical on such freeways in China.

The experiment is based on the simulated road scene model, which is designed by the interchange of the freeway in Shandong China. It has a good horizontal and vertical alignment, and the cross slope is 2%, which is consistent with the requirements of national design codes [33]. The experimental research road section is divided into three sections. The first section is an adaptation road section. It is an ordinary road with a length of 1000m and a speed limit of 60km/h. The second section is a ramp connecting the adaptation section and the main lane of the freeway. The radius and length of the ramp are in compliance with design specifications. The speed limit is 40km/h. The third section is the main lane of the freeway. The speed limit of outer two lanes is 100km/h, and the limit of inner two lanes is 120km/h.

The third section is divided into two sections. The first section is the adaptation section of the vehicle after the acceleration lane enters the main section, and the length is 1000m. The second section is the research section of the central divider with an opening. At the beginning and the end point, the corresponding chevron guideline and the notice mark are set at the standard position. The length of the opening is one of the experimental control independent variables.

### 3.2 Apparatus and participants

**Fig 2:**
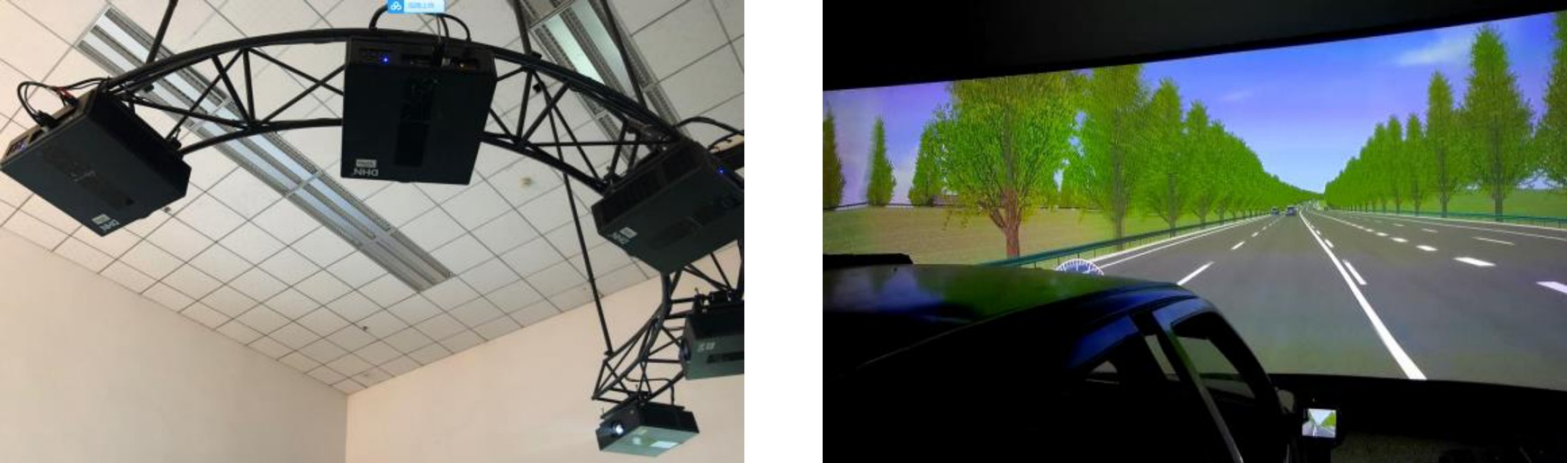
The simulated driving experimental platform.

This study uses a simulated driving experimental platform based on a real vehicle, which is located in the Intelligent Transport Systems Center of Wuhan University of Technology, as shown in Fig 2. It consists of a five-channel projector and three rearview mirrors, as well as a fusion device and multi-channel cluster simulation software. In order to improve the realism, a completely real vehicle was placed, which has a real force feedback system, including the throttle, brake pedal, steering wheel and the like. The test road model was built with the UC-win/Road Ver.13 developed by Forum 8 Company in Japan, and the vehicle data was collected through the software, CAN card and self-developed plug-ins. The sampling interval was 0.05 seconds (the actual data period used was 0.1s). The data information required for this study mainly includes vehicle speed, driving time, vehicle position, lane offset, distance between the test vehicle and the preceding vehicle and so on.

In this study, 25 participants were recruited, including 22 males and 3 females. We have indicated to all participants that all their driving data and their basic personal information were available and the consent was informed. All participants signed the notification agreement. The age range of the participants was 24 to 56 years old, with a mean age of 32.9. The driving age range of the participants was 2 to 20 years, with a mean of 7.0. The driving mileage range of the participants was 2,000 to 3,000,000 km, with a mean mileage of 388,940. Among them are 10 non-professional drivers, mainly recruited postgraduate students; and 15 professional drivers, mainly taxi drivers. For the purpose of this study, the driving behavior under the influence of opening length and traffic flow was mainly explored, so the gender was not controlled.

### 3.3 Research procedure

In order to meet certain requirements and achieve consistent results, the research team developed the following experimental procedures. We had issued consent to all participants, and there were no minors among the participants. The process was reviewed by a research team academic committee.

(1) Inform the participant of the possible physiological effects. If there is discomfort or vertigo symptoms, the participant can ask to stop the experiment immediately. The participant is required to drive according to the real intention, not to deliberately hit the guardrail or attempt to collide with other vehicles.

(2) Inform the basic situation of the simulated driving, and introduce the experimental scene and the requirements. The participant is required to fill in the basic information form and the commitment letter.

(3) After familiarizing with the driving simulator (about 20 minutes), the simulated driving test was officially started. A laboratory assistant was provided throughout the test to guide the route of driving but did not interfere with the usual driving habits of the participant, allowing the participant to maintain a natural driving state.

(4) The driving task of the subject was to enter the ramp from the ordinary road, then enter the acceleration lane, and enter the main line. After passing through the adapted section with a length of 1000m, the vehicle entered the research section and changed lanes at the opening of the median strip with different lengths into the inner two lanes. The test ends after the vehicle has completely driven through the research section.

(5) At the end of each test, the participant is required to take a break for a period.

(6) In order to avoid the memory effect on the test road, the 9 types of test scenarios were carried out in random order, and that every two adjacent test road scenes were different in terms of traffic volume and opening length of central divider.

**Fig 3:**
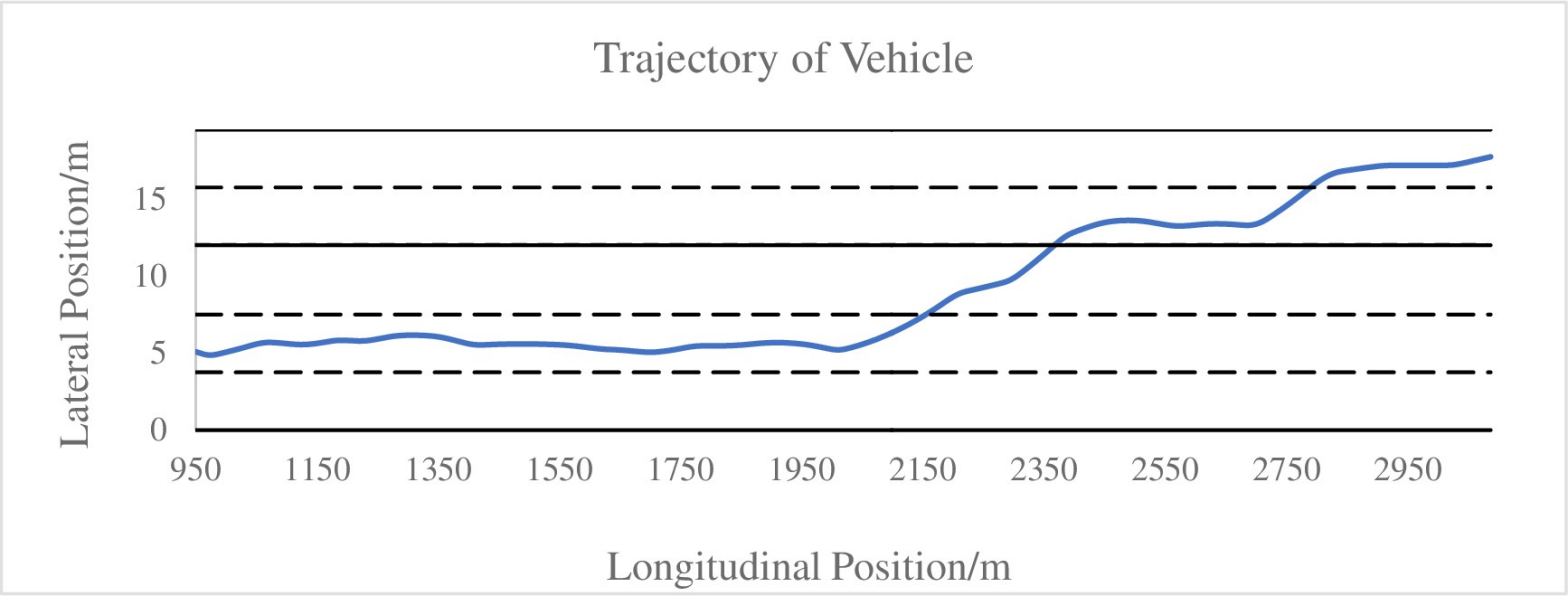
The vehicle driving trajectory diagram of a simulated driving test.

Each of the participants drove 9 scenarios and 225 valid test data was received. After pre-processing screening, subsequent processing is performed. Fig 3 is a vehicle driving trajectory diagram of a simulated driving test obtained after pre-treatment. The starting point of the valid data is at the point where the vehicle has officially entered the main line, at the position of 950m from the acceleration lane. After 1000m of the adaptation section, the vehicle can start to enter the inner lane at the position of 1950m through the opening section of the median strip.

## 4. Results

### 4.1 Speed profiles

The speed change process of the vehicle is as follows: the vehicle on the ramp enters into the main lane of the freeway after accelerating in the acceleration lane, then drives at the outer lane at a speed limit of 100 km/h for 1 km to stabilize the driving speed, and then changes into the inner lane through the opening section of the median strip, with speed changing according to personal driving habits, and finally drives stably at a speed limit of 120 km/h on the inner lane. The stability of speed is a manifestation of driving safety. Unstable speed has a certain relationship with traffic accidents [34] [35] [36]. In order to facilitate the study of the change of vehicle speed, this study considers the experimental road as a section every 100m, to study the continuous variation of vehicle speed on different sections. The total length of the experimental section with an opening length of 800m is 2138m, and the number of sections is 21. The total length of the experimental section with an opening length of 1200m is 2538m, and the number of sections is 25. The total length of the experimental section with an opening length of 1700m is 3038m, and the number of sections is 30. In this study, ANOVA was used to analyze the factors affecting the speed. The dependent variable is the average speed of each section. The independent variables are the length of the OSMS and the traffic flow. The descriptive statistic results are shown in Table 1, and the ANOVA results are shown in Table 2.

**Table 1:** Descriptive statistic results of driving speed.

Opening Length(m)	Traffic Flow(pcu/d)	N	Speed(Mean)	Speed(SD)
800	23000	21	110.1	5.6
	28000	21	103.8	4.7
	33000	21	109.3	4.7
1200	23000	25	101.3	7.7
	28000	25	111.3	5.4
	33000	25	109.8	5.8
1700	23000	30	113.1	5.2
	28000	30	111.3	6.1
	33000	30	111.5	7.3

**Table 2:** ANOVA results of driving speed.

Source	d.f.	F-Ratio	Sig.
Opening Length	2	14.684	0.000***
Traffic Flow	2	2.224	0.111
Opening Length* Traffic Flow	4	12.995	0.000***

The results show that the effect of opening length on vehicle speed is significant (F = 14.684, p = 0.000). When the opening length is 800m, the average speed is 107.7km/h. When the opening length is 1200m, the average speed is 107.5km/ h. When the opening length is 1700 m, the average speed of the vehicle is 112.0 m. The impact of traffic flow on vehicle speed was not significant (F = 2.224, p = 0.111). There are significant interactions between the variables (F = 12.995, p = 0.000), as listed in Table 3. The interaction effect is that under low traffic flow and moderate flow conditions, the opening length significantly affects the average speed, and the longer the opening length, the faster the average speed. However, when the traffic flow is at a high level, the opening length does not have a significant impact on the average speed. There are some previous research about the traffic flow effect on the speed when driving on a freeway, and similar studies have shown that the emergence of a traffic jam could cause low speed of traffic [37] [38]. This may be due to the fact that when the traffic volume is large, the vehicle is greatly affected by the traffic flow from the surrounding, and the speed of the vehicle is subject to stricter restrictions on the relatively long OSMS.

**Table 3:** Simple effect analysis results of driving speed.

Traffic Flow		Sum of Squares	d.f.	Mean Square	F	Sig.
23000	Contrast	1982.601	2	991.301	27.583	0.000*
	Error	7870.529	219	35.938		
28000	Contrast	868.350	2	434.175	12.081	0.000*
	Error	7870.529	219	35.938		
33000	Contrast	72.492	2	36.246	1.009	0.366
	Error	7870.529	219	35.938		

### 4.2 Maximum deceleration

Studies have shown that the maximum deceleration in the longitudinal direction of the vehicle reflects the limit of vehicle braking and is a direct measure of the driver's longitudinal driving comfort [39] [40]. Descriptive statistics and ANOVA analysis for the maximum deceleration (ACC_max_) of the vehicle appearing in each test are shown in Tables 4 and 5.

**Table 4:** Descriptive statistic results of the maximum deceleration.

Opening Length(m)	Traffic Flow(pcu/d)	N	ACC_max_ (Mean)	ACC_max_ (SD)
800	23000	25	-1.10	0.60
	28000	25	-1.18	0.87
	33000	25	-1.41	1.09
	SUM	75	-1.23	0.87
12000	23000	25	-1.67	1.13
	28000	25	-1.14	0.58
	33000	25	-1.41	0.77
	SUM	75	-1.41	0.87
17000	23000	25	-1.80	1.54
	28000	25	-1.28	1.09
	33000	25	-1.29	0.73
	SUM	75	-1.46	1.18
SUM	23000	75	-1.52	1.18
	28000	75	-1.20	0.86
	33000	75	-1.37	0.87

**Table 5:** ANOVA results of the maximum deceleration.

Source	d.f.	F-Ratio	Sig.
Opening Length	2	1.106	0.333
Traffic Flow	2	2.038	0.133
Opening Length* Traffic Flow	4	1.363	0.248

**Fig 4:**
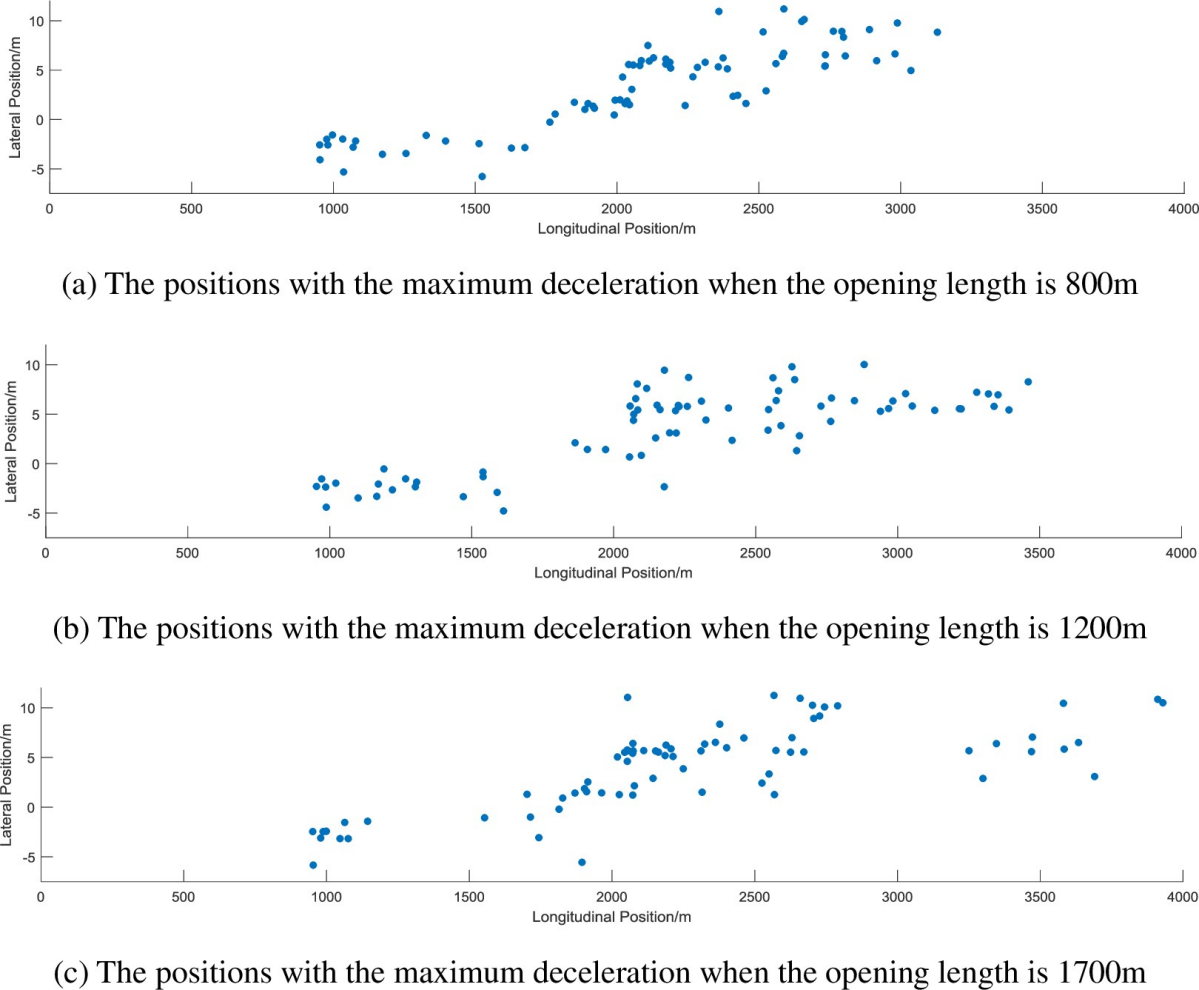
The positions of vehicles with the maximum deceleration rates under different opening lengths.

It can be seen from the above results that both the opening length (F = 1.106, p = 0.333) and the traffic flow (F = 2.038, p = 0.133) have no significant effect on the maximum deceleration of the vehicle in the OSMS section, and there is no interaction effect (F = 1.363, p = 0.248). This shows that the opening length of the OSMS and the traffic flow have no significant effect on the longitudinal deceleration of the vehicle. In order to study the change of the deceleration willingness of the driver in the OSMS section, this study further statistically analyzes the position of the vehicle when the driving reaches the maximum braking deceleration in each test under different opening length and traffic flow. The positions of the vehicles with the maximum braking deceleration under the three opening lengths are plotted as 3 scatter plots, as shown in Fig 4.

It can be initially found from the scatter plots that as the opening length increases, the concentration point at which the vehicle adopts the maximum deceleration is closer to the middle of the opening section. In order to further verify the effect of the opening length and the traffic flow on the maximum deceleration, a statistical analysis is performed on the position of the vehicle when the maximum braking deceleration is reached in each test, which is divided into the longitudinal position (X_1_) and lateral position (Y_1_). Descriptive statistics and ANOVA results are shown in Tables 6 and 7.

**Table 6:** Descriptive statistic results of the position with the maximum deceleration.

Opening Length(m)	Traffic Flow(pcu/d)	N	X_1_ (Mean)	X_1_ (SD)	Y_1_ (Mean)	Y_1_ (SD)
800	23000	25	1850.09	633.47	1.98	4.04
	28000	25	2178.40	457.30	3.79	4.48
	33000	25	2248.84	599.46	4.12	4.23
	SUM	75	2092.44	587.47	3.30	4.30
12000	23000	25	2222.34	701.94	3.35	4.32
	28000	25	2279.23	711.57	3.82	4.28
	33000	25	2193.19	684.21	3.30	3.50
	SUM	75	2231.59	690.75	3.49	4.00
17000	23000	25	2137.76	687.82	3.68	4.17
	28000	25	2483.02	864.82	4.76	4.09
	33000	25	2140.33	602.57	3.88	4.57
	SUM	75	2253.70	735.13	4.11	4.25
SUM	23000	75	2070.07	684.92	3.00	4.19
	28000	75	2313.55	700.63	4.12	4.25
	33000	75	2194.12	623.00	3.77	4.08

**Table 7:** ANOVA results of the position with the maximum deceleration.

Source	d.f.	X_1_ (F-Ratio)	X_1_ (Sig.)	Y_1_ (F-Ratio)	Y_1_ (Sig.)
Opening Length	2	1.283	0.279	0.766	0.466
Traffic Flow	2	2.488	0.085	1.383	0.253
Opening Length* Traffic Flow	4	1.180	0.321	0.541	0.706

It can be seen from the above results that the length of the OSMS and the traffic flow have no significant influence on the corresponding longitudinal and lateral position of vehicle when the driver takes the maximum brake deceleration. The longitudinal deceleration can reflect the stability and comfort of the vehicle while driving on the road, because frequent deceleration causes the driver to withstand longitudinal forces throughout the driving process, causing longitudinal discomfort [41]. This result once again verifies that the length of the OSMS and the traffic flow have no significant impact on driving comfort. It also shows that when designing the OSMS for road capacity expansion project with one-way flow freeway, driving safety is a priority, and less affected driving comfort may not be a primary consideration.

### 4.3 Safety surrogate indicators

(1) Minimum Time Headway

It has been proved that the Time Headway (THW) can reflect the time distance between the vehicle driven and the vehicle in front, and the minimum value can represent the safety margin of each driving [42] [43]. This study analyzes the minimum value of THW for each driving. Table 8 is a descriptive statistical result of the mean values (THW_ave_) and minimum (THW_min_) of THW, and Table 9 is the ANOVA results.

**Table 8:** Descriptive statistic results of the minimum THW.

Opening Length(m)	Traffic Flow(pcu/d)	N	THW_ave_ (Mean)	THW_ave_ (SD)	THW_min_ (Mean)	THW_min_ (SD)
800	23000	25	4.86	0.36	3.24	1.53
	28000	25	4.49	0.68	2.72	1.75
	33000	25	4.68	0.95	2.33	1.38
	SUM	75	4.68	0.71	2.76	1.58
12000	23000	25	4.80	0.52	3.34	1.58
	28000	25	4.57	0.46	2.15	1.34
	33000	25	4.57	0.60	2.54	1.55
	SUM	75	4.65	0.53	2.68	1.56
17000	23000	25	4.59	0.55	2.67	1.66
	28000	25	4.54	0.65	2.75	1.65
	33000	25	4.48	0.63	2.21	1.56
	SUM	75	4.54	0.61	2.54	1.62
SUM	23000	75	4.75	0.49	3.08	1.60
	28000	75	4.53	0.60	2.54	1.59
	33000	75	4.58	0.74	2.36	1.48

**Table 9:** ANOVA results of the minimum THW.

Source	d.f.	THW_ave_ (F-Ratio)	THW_ave_ (Sig.)	THW_min_ (F-Ratio)	THW_min_ (Sig.)
Opening Length	2	1.029	0.359	0.368	0.692
Traffic Flow	2	2.568	0.079	4.376	0.014*
Opening Length* Traffic Flow	4	0.523	0.719	1.214	0.306

**Fig 5:**
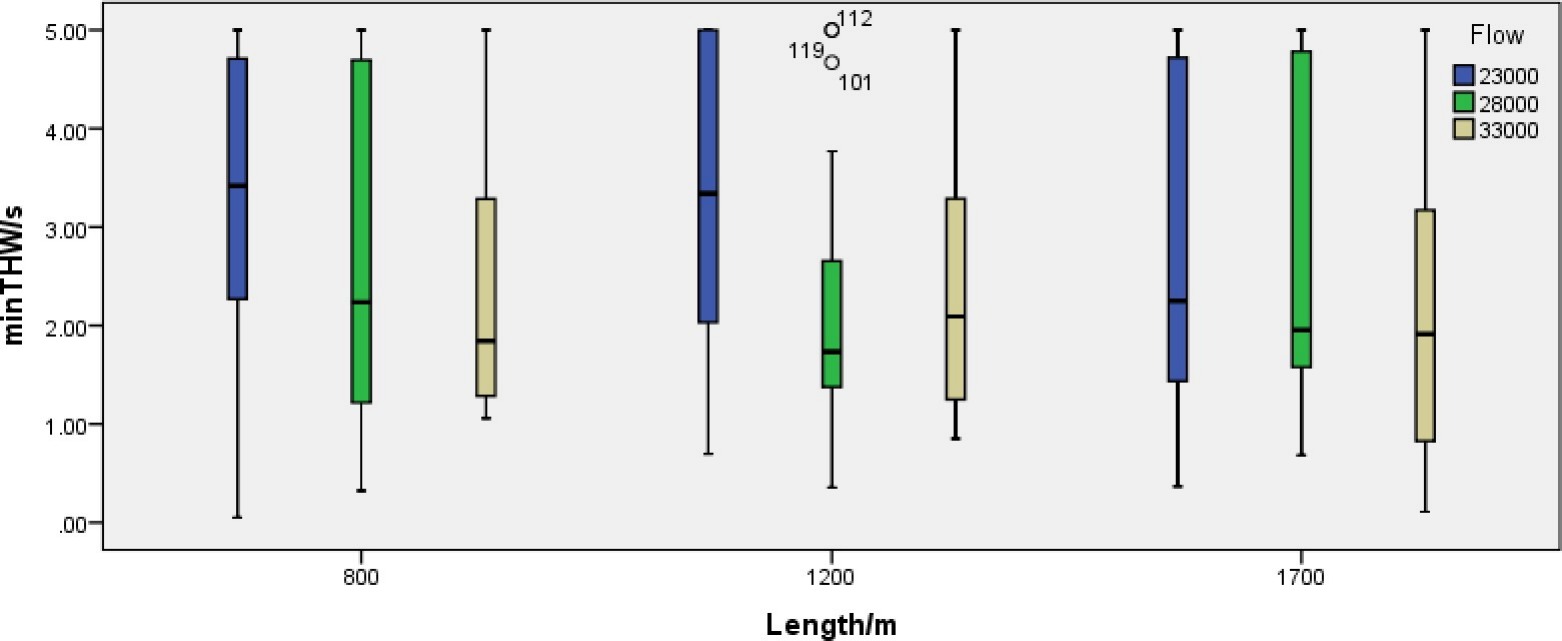
The minimum THW with different opening lengths and traffic flow.

The results show that the impact of traffic flow on the minimum THW is significant (F = 4.376, p = 0.014). When the traffic flow is low, the average of the minimum THW is 3.08s. When the traffic flow is moderate, the average of the minimum THW is 2.54s. When the traffic flow is high, the average of the minimum THW is 2.36s. The results are shown in Fig 5. The effects of the opening length on the average and minimum THW are not significant. No significant interactions were found between the variables.

**Fig 6:**
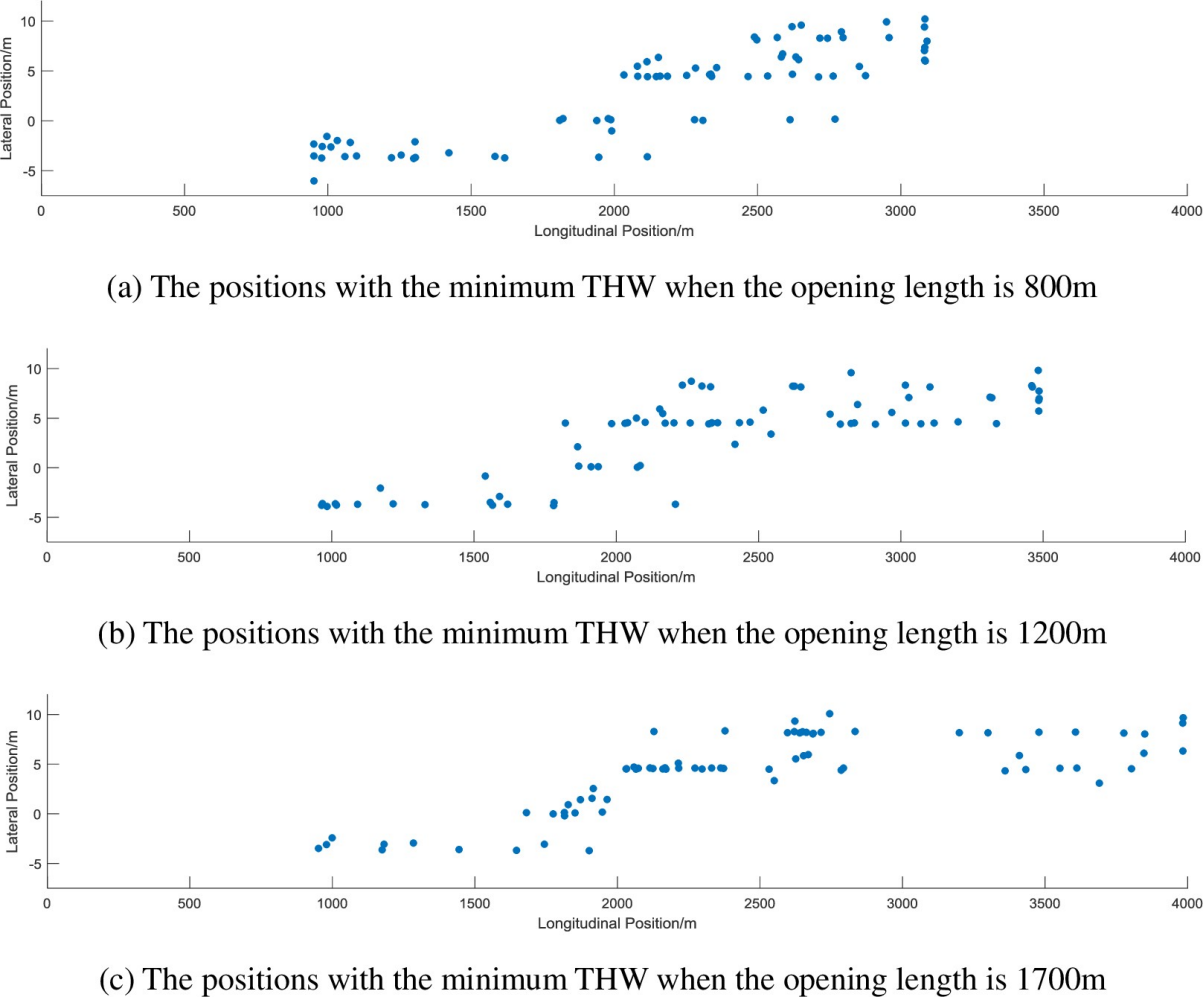
The positions of vehicles with the minimum THW under different opening lengths.

In order to further analyze the minimum THW, this study made 3 scatter plots of the position of the vehicle with the minimum THW in each test of different opening length, as shown in Fig 6.

It is shown from Fig 6 that when the length of the OSMS increases, the position of the vehicles with the minimum THW gradually advances. The position is divided into the longitudinal position (X_2_) and lateral position (Y_2_). Further analysis of the vehicle position corresponding to the minimum THW shows that the opening length has a significant influence on the longitudinal position. The results are shown in Tables 10 and 11.

**Table 10:** Descriptive statistic results of the position with the minimum THW.

Opening Length(m)	Traffic Flow(pcu/d)	N	X_2_(Mean)	X_2_(SD)	Y_2_(Mean)	Y_2_(SD)
800	23000	25	2076.07	780.80	2.12	4.81
	28000	25	2266.24	593.51	4.06	4.27
	33000	25	2109.73	639.26	2.37	4.74
	SUM	75	2150.68	671.91	2.85	4.63
12000	23000	25	2128.04	747.55	2.31	4.37
	28000	25	2408.08	625.39	3.72	4.40
	33000	25	2483.43	747.06	4.07	3.96
	SUM	75	2339.85	716.09	3.37	4.26
17000	23000	25	2287.48	760.05	3.04	3.71
	28000	25	2460.35	837.33	3.77	3.80
	33000	25	2658.96	728.08	5.55	3.81
	SUM	75	2468.93	781.03	4.12	3.87
SUM	23000	75	2163.86	757.97	2.49	4.28
	28000	75	2378.23	689.42	3.85	4.11
	33000	75	2417.37	733.92	4.00	4.33

**Table 11:** ANOVA results of the position with the minimum THW.

Source	d.f.	X_2_ (F-Ratio)	X_2_ (Sig.)	Y_2_ (F-Ratio)	Y_2_ (Sig.)
Opening Length	2	3.690	0.027*	1.722	0.181
Traffic Flow	2	2.682	0.071	2.900	0.057
Opening Length* Traffic Flow	4	0.577	0.680	1.108	0.354

The results show that the effect of the opening length on the longitudinal position of the vehicle with the minimum THW is significant (F = 3.690, p = 0.027). When the opening length is 800m, the average longitudinal position is 2150.68m. When the opening length is 1200m, the average longitudinal position is 2339.85m. When the opening length is 1700m, the average longitudinal position is 2468.93m. The impact of traffic flow on the position is not significant. No significant interactions were found between the variables.

The significant effect of the opening length on the longitudinal position with the minimum THW may be due to changes in the opening length, as well as changes in the spatial constraints, so that the longitudinal position of the vehicle may also change. On the other hand, as can be seen in conjunction with Fig 6, when the opening length is 800m, vehicles are more generally concentrated at the end of the opening section with minimum THW, which is gradually alleviated as the opening length increases. The minimum THW represents the safety margin of the vehicle. This concentration occurs at the end of the opening section and is likely to lead to an increase in road safety risks. The possible reason is that when the opening section is too short, the drivers have no time to adapt, and eventually start decelerating at the end of the opening section in a bid to change into the inner lane. At this time, the timing of the lane change is tight, and the short distance is more likely to occur with the preceding vehicle, resulting in a phenomenon wherein the minimum THW is unsafe. It is also similar to the conclusion that the merging vehicle could become aggressive in order to complete the merging maneuver over the elapsed time, even if it has high safety risk with respect to the through vehicles [44] [45].Therefore, it is worth noting that the safety margin of the vehicle at the end of the opening section is reduced due to the short opening length. In order to alleviate this situation, on the one hand, the opening length can be appropriately increased, and on the other hand, the traffic sign can be used to remind the driver to change lanes as early as possible.

(2) Maximum Reciprocal of Time to Collision

It has been proved that when the speed of the rear vehicle is greater than that of the preceding vehicle, the time to collision (TTC) can represent the relative speed and relative distance between the vehicle and the vehicle in front, which needs to be considered during the lane change [46] [47] [48]. The TTC indicator of a vehicle *i-1* at instant time with respect to a vehicle in front *i* can be expressed by:

$$TTC=\frac{X_{i}-X_{i-1}-l}{V_{i-1}-V_{i}}\;(V_{i-1}>V_{i})$$ (1)

where *l* denotes the length of vehicle, *X* the position, and *V* the speed.

Some new safety indicators based on the TTC are suitable for traffic safety analyses [49]. For example, the time exposed time-to-collision indicator (TET) is a summation of all moments (over the considered time period) that a vehicle approaches a front vehicle with a TTC below the threshold value TTC*. On this basis, the time integrated time-to-collision indicator (TIT) is a time integral which uses the integral of the TTC profile of drivers to express the level of safety. It can take the impact of the TTC-value as well as the time into account in the safety assessment.

However, crashes do not occur frequently on freeway, and the difference in driving speed between vehicles is not large, so the TTC calculated from the above Formula (1) is relatively discrete, and its value is relatively large because the denominator is very small (the result of *V*_*i-1*_-*V*_*i*_ is very small), which is difficult to count. This can make it difficult to accurately calculate both TET and TIT indicators. Therefore, this study uses TTC_i_ (the reciprocal of TTC) to study the time to collision between vehicles, and its maximum value can represent the safety margin. The TTC_i_ indicator of a vehicle *i-1* at instant time with respect to a vehicle in front *i* can be expressed by:

$$TTC_{i}=\frac{V_{i-1}-V_{i}}{X_{i}-X_{i-1}-l}\;(V_{i-1}>V_{i})$$ (2)

where *l* denotes the length of vehicle, *X* the position, and *V* the speed.

It can be considered that the lower the value of TTC_i_, the safer the situation, because the relative speed between the subject vehicle and the vehicle in front is smaller and the relative distance is longer. Table 12 shows the descriptive statistical result of the average (TTC_i ave_) and maximum (TTC_i max_) values of TTC_i_, and the ANOVA results are shown in Table 13.

**Table 12:** Descriptive statistic results of the TTC_i_.

Opening Length(m)	Traffic Flow(pcu/d)	N	TTC_i ave_ (Mean)	TTC_i ave_ (SD)	TTC_i max_ (Mean)	TTC_i max_ (SD)
800	23000	25	0.042	0.070	5.59	27.33
	28000	25	0.034	0.030	0.15	0.09
	33000	25	0.049	0.049	0.16	0.07
	SUM	75	0.042	0.052	1.97	15.78
12000	23000	25	0.023	0.019	0.17	0.06
	28000	25	0.045	0.031	0.19	0.06
	33000	25	0.038	0.040	0.16	0.09
	SUM	75	0.035	0.032	0.17	0.07
17000	23000	25	0.032	0.036	0.15	0.11
	28000	25	0.046	0.049	0.16	0.07
	33000	25	0.043	0.036	0.61	2.08
	SUM	75	0.040	0.041	0.30	1.21
SUM	23000	75	0.032	0.047	1.97	15.78
	28000	75	0.042	0.038	0.17	0.07
	33000	75	0.043	0.041	0.31	1.21

**Table 13:** ANOVA results of the TTC_i_.

Source	d.f.	TTC_i ave_ (F-Ratio)	TTC_i ave_ (Sig.)	TTC_i max_ (F-Ratio)	TTC_i max_ (Sig.)
Opening Length	2	0.482	0.618	0.898	0.409
Traffic Flow	2	1.503	0.225	0.904	0.406
Opening Length* Traffic Flow	4	0.952	0.435	1.031	0.392

It is shown that the opening length and the traffic flow have no significant effect on the average and maximum values of TTC_i_, and there is no interaction effect. The study further statistically analyzes the corresponding vehicle position when the maximum TTC_i_ appears, which is divided into the longitudinal (X_3_) and lateral position (Y_3_). The results are shown in Table 14 and Table 15.

**Table 14:** Descriptive statistic results of the position with maximum TTC_i_.

Opening Length(m)	Traffic Flow(pcu/d)	N	X_3_(Mean)	X_3_(SD)	Y_3_(Mean)	Y_3_(SD)
800	23000	25	49.39	246.96	-0.15	0.76
	28000	25	107.63	538.17	0.34	1.69
	33000	25	0.00	0.00	0.00	0.00
	SUM	75	52.34	340.11	0.06	1.07
12000	23000	25	152.62	542.21	0.18	1.82
	28000	25	177.12	625.72	0.17	1.83
	33000	25	111.22	388.24	-0.30	1.03
	SUM	75	146.99	521.50	0.02	1.60
17000	23000	25	46.99	234.94	-0.15	0.73
	28000	25	0.00	0.00	0.00	0.00
	33000	25	412.74	966.64	1.17	2.80
	SUM	75	153.24	596.19	0.34	1.75
SUM	23000	75	83.00	368.09	-0.04	1.21
	28000	75	94.92	475.71	0.17	1.42
	33000	75	174.65	618.67	0.29	1.81

**Table 15:** ANOVA results of the position with maximum TTC_i_.

Source	d.f.	X_3_ (F-Ratio)	X_3_ (Sig.)	Y_3_ (F-Ratio)	Y_3_ (Sig.)
Opening Length	2	0.991	0.373	1.066	0.346
Traffic Flow	2	0.770	0.464	0.966	0.382
Opening Length* Traffic Flow	4	2.461	0.046*	3.312	0.012*

The results show that both the opening length and the traffic flow have no significant effect on the longitudinal and lateral position of the vehicle corresponding to the maximum TTC_i_, but there is a simple interaction between them. The simple interaction effects of the opening length and traffic flow are analyzed respectively, and the results are shown in Table 16 and Table 17.

**Table 16:** Simple effect analysis results of the longitudinal position with maximum TTC_i_.

Traffic Flow		Sum of Squares	d.f.	Mean Square	F	Sig.
23000	Contrast	181845.183	2	90922.592	0.376	0.687
	Error	52235012.423	216	241828.761		
28000	Contrast	398206.031	2	199103.015	0.823	0.440
	Error	52235012.423	216	241828.761		
33000	Contrast	2280295.886	2	1140147.943	4.715	0.010*
	Error	52235012.423	216	241828.761		

**Table 17:** Simple effect analysis results of the lateral position with maximum TTC_i_.

Traffic Flow		Sum of Squares	d.f.	Mean Square	F	Sig.
23000	Contrast	1.788	2	0.894	0.413	0.662
	Error	467.445	216	2.164		
28000	Contrast	1.425	2	0.712	0.329	0.720
	Error	467.445	216	2.164		
33000	Contrast	30.067	2	15.034	6.947	0.001*
	Error	467.445	216	2.164		

The results show that when the traffic flow is at a low or moderale level, the opening length has no significant effect on the longitudinal and lateral positions of the vehicle corresponding to the maximum TTC_i_. However, when the traffic flow is high, the opening length significantly affects the two positions, and the longer the opening length, the longer the longitudinal position, and the closer the lateral position is to the inner lane. This may be because in high-density traffic conditions, traffic is denser and vehicles are more prone to crash. However, in general, the average longitudinal position in all tests is relatively close to the beginning point. This indicates that the vehicles are not prone to crash in the opening section. It is also generally possible for drivers to control the distance and speed difference from the preceding vehicle when negotiating a lane change, into the inner lanes. The focus on increasing the safety of lane change has a significant impact on lowering the occurrence of crashes [50].

## 5. Conclusion

In order to study the driving performance at the OSMS on road capacity expansion project with one-way flow of freeway, this study separately controlled 9 different simulation experimental scenarios of opening length and traffic flow. 25 participants were recruited to perform 225 simulated driving tests using the driving simulator, and a comprehensive analysis of the obtained test data was conducted to study the driving characteristics. The conclusions are as follows.

(1) The effect of the length of the opening section on the speed of the vehicle is significant and there is an interaction with the traffic flow. When the traffic flow is low or moderate, the opening length can significantly affect the speed of the vehicle. However, when the traffic flow is at a high level, the opening length does not have a significant impact on the average speed. This shows that when the traffic flow is low, the influence of the length of the opening section on the vehicle speed should be fully considered. When the traffic flow is high, the length of the opening section cannot be regarded as an important consideration for the vehicle speed control management. The length of the opening section and the traffic flow have no significant effect on the maximum values of deceleration of the vehicle. This shows that the driving comfort at the OSMS is not easily affected by them. When considering this type of road design, comfort can be an inferior priority to safety.

(2) The impact of traffic flow on the minimum values of THW is significant. The higher the traffic flow, the smaller the minimum values of THW, especially when the traffic flow is high, the values of THW is gradually decreasing as the opening length increases. This shows that in the case of large traffic volume on the freeway merely extending the length of the opening section does not necessarily optimize the safety of the road when designing this type of road. It can be considered that the traffic density can be reduced by improving the traffic management method, and the appropriate traffic control devices can be used to remind the drivers to change lanes safely, as early as possible and control the distance between other vehicles.

(3) The effect of the opening length on the longitudinal position of the vehicle corresponding to the minimum values of THW is significant. As the length of the opening decreases, the vehicles are more generally concentrated at the end of the opening area with the minimum values of THW. This situation is likely to lead to an increase in road safety risks. The reason may be that the length of the opening section is too short, the driver has no time to adapt, and eventually initiates deceleration and lane at the end of the OSMS. The timing of the lane change is tight, and the short vehicle distance is more likely to occur with the preceding vehicle, resulting in a phenomenon wherein the minimum values of THW are reached.

(4) Both the opening length and the traffic flow have no significant effect on the longitudinal and lateral position of the vehicle corresponding to the maximum values of TTC_i_, but there is a simple interaction between them. When the traffic flow is at a low or moderate density, the opening length has no significant effect on the longitudinal and lateral position. However, when the traffic volume is high, the opening length significantly affects both locations. Overall, the average longitudinal position of the vehicle corresponding to the maximum values of TTC_i_ is relatively close to the beginning point of the opening section, which indicates that the drivers can generally control the distance and speed difference from the preceding vehicle when changing the lane from the opening section to the inner lane.

In general, the conclusions of this study have a good reference value for the application of road capacity expansion project with one-directional flow freeway. When designing the opening section, it is necessary to consider the economics of the project in conjunction with the safety of the driving environment. When the opening section is too short, the reaction demand for the drivers is increased, and the driving risk is increased. However, the long opening section not only increases the engineering cost, but also may cause the drivers to travel longer on the opening section, which also increases the risk of driving. In addition, when the traffic volume is high, the impact of the opening length on driving performance will be relatively weakened to some extent. Therefore, it is recommended that in the application, the actual traffic density of the road should be carefully considered, and the moderate length of the opening section should be reasonably designed, rather than simply calculating the road geometry according to the design code specifications.

The current conclusions of this study are mainly limited to the driving behavior at the entrance of the OSMS section of the freeway, but it is also foreseen that for the exit area, the length of the opening section and the traffic flow will also affect the above driving performance to some extent. It is suggested to extend the research on the driving behavior at the OSMS to the exit area in the follow-up study, increase the number of participants, and conduct further analyses based on field operational test data.
